# Hard X-ray single-shot spectrometer of PAL-XFEL

**DOI:** 10.1107/S1600577524009779

**Published:** 2025-01-01

**Authors:** Sangsoo Kim, Jae Hyuk Lee, Daewoong Nam, Gisu Park, Myong-jin Kim, Intae Eom, Inhyuk Nam, Chi Hyun Shim, Jangwoo Kim

**Affiliations:** a Pohang Accelerator Laboratory/POSTECH, 80 Jigokro-127-beongil, Pohang, Gyeongbuk37673, Republic of Korea; b Department of Physics Ulsan National Institute of Science and Technology Ulsan44919 Republic of Korea; RIKEN SPring-8 Center, Japan

**Keywords:** XFEL, spectrometer, PAL-XFEL

## Abstract

A transmissive single-shot spectrometer has been developed to monitor shot-to-shot spectral structures in the hard X-ray beamline of the Pohang Accelerator Laboratory X-ray Free Electron Laser (PAL-XFEL). The established spectrometer comprises 10 µm-thick Si crystals bent to a radius of curvature of 100 mm. Depending on the photon energy range, either the Si (111) or Si (110) crystal can be selected for spectral analysis. Especially in the energy range 4.5–17 keV, the spectrometer is designed to cover a spectral range wider than the full free-electron laser bandwidth and to guarantee a high resolution sufficient for resolving each spectral spike. This paper presents the design specifications, instruments and performance of this spectrometer, which has also been applied to demonstrate the spectral properties of various XFEL sources, such as self-amplified spontaneous emission, monochromatic and seeded beams.

## Abstract 2

A hard X-ray single-shot spectrometer comprising thin, bent Si crystals has been developed for the Pohang Accelerator Laboratory X-ray Free-Electron Laser (XFEL), for detailed analysis of ultrafast 4.5–17 keV XFEL pulses with a high spectral resolution. This instrument facilitates shot-to-shot spectral structure monitoring and optimization of the operating conditions of the XFEL owing to its ability to provide comprehensive data on the spectral properties and fluctuations of self-amplified spontaneous emission, monochromatic and seeded XFEL modes.

## 1. Introduction

With the advent of X-ray free-electron lasers (XFELs), extremely intense ultrashort X-ray pulses have facilitated the exploration of ultrafast XFEL science through single-shot measurements. These sources are primarily based on the self-amplified spontaneous emission (SASE) process, whose onset is triggered by the random shot noise of an electron bunch (Bonifacio *et al.*, 1984 ▸; Saldin *et al.*, 2000 ▸; Huang & Kim, 2007 ▸). Therefore, the inevitable stochastic nature of SASE radiation results in strong fluctuations in both spectral intensity and distributions. Furthermore, proper interpretation of the spectral structures in a SASE spectrum developed throughout the SASE process, from the initialization of the electron bunch to SASE saturation, provides insights into the XFEL parameters; for example, the width of the spectral spikes is correlated with the pulse duration (Shastri *et al.*, 2001 ▸; Inubushi *et al.*, 2012 ▸). Increasing the pulse duration shortens the width of the spectral spikes and vice versa. The spectral distribution determines the photon energy, from which the electron beam energy can be evaluated with high accuracy. Therefore, a single-shot spectrometer is essential for both the analysis of experimental data and optimization of SASE parameters.

Several techniques have been developed at different XFEL facilities to characterize the shot-to-shot spectral structures observed in the hard X-ray regime. For the SPring-8 Ångström Compact free electron LAser (SACLA), a flat crystal analyzer with an elliptical focusing mirror has been designed as a single-shot spectrometer (Yabashi *et al.*, 2006 ▸; Inubushi *et al.*, 2012 ▸; Katayama *et al.*, 2013 ▸). Although this method can achieve a high spectral resolution, the focusing mirrors present in the system interrupt the tandem experiment downstream of the spectrometer. Thus, non-invasive techniques such as the thin, bent crystal method (Zhu *et al.*, 2012 ▸; Zhu *et al.*, 2013 ▸; Rich *et al.*, 2016 ▸) and transmission grating method (Karvinen *et al.*, 2012 ▸) have been commissioned at the Linac Coherent Light Source. A previous study suggests that a bent crystal spectrometer, which combines the advantages of both methods, can be placed in the path of the first-order diffracted beam of a transmission diamond grating setup (Makita *et al.*, 2015 ▸). This combined technique is useful even at other XFEL facilities, such as the European XFEL (Kujala *et al.*, 2020 ▸) and SwissFEL (David *et al.*, 2021 ▸). Another combined technique that integrates a transmission grating with a focusing mirror and flat crystal has also been developed at SACLA (Katayama *et al.*, 2016 ▸). In addition, many other methods have been developed to characterize the single-shot spectra of SASE beams (Kohn *et al.*, 2013 ▸; Katayama *et al.*, 2013 ▸; Brenner *et al.*, 2019 ▸; Inoue *et al.*, 2022 ▸).

Among these techniques, the thin, bent crystal method has been adopted for a hard X-ray single-shot spectrometer in the Pohang Accelerator Laboratory X-ray Free Electron Laser (PAL-XFEL) facility. This paper introduces the instrumentation and spectral data for characterizing the performance of this instrument.

## 2. Design and instrumentation

The PAL-XFEL can provide XFEL pulses at a maximum repetition rate of 60 Hz, and the pulse energy of the SASE beam is greater than 1.2 mJ in the hard X-ray regime (Eom *et al.*, 2022 ▸). Because of the relatively low repetition rate, the thermal load on the bent crystal appears to be less severe, although it is not negligible. Moreover, the transmission grating method cannot be implemented owing to the extremely narrow space available for a single-shot spectrometer. Therefore, to overcome these limitations, we developed a hard X-ray single-shot spectrometer that utilizes thin, bent Si crystals.

**Figure 1:**
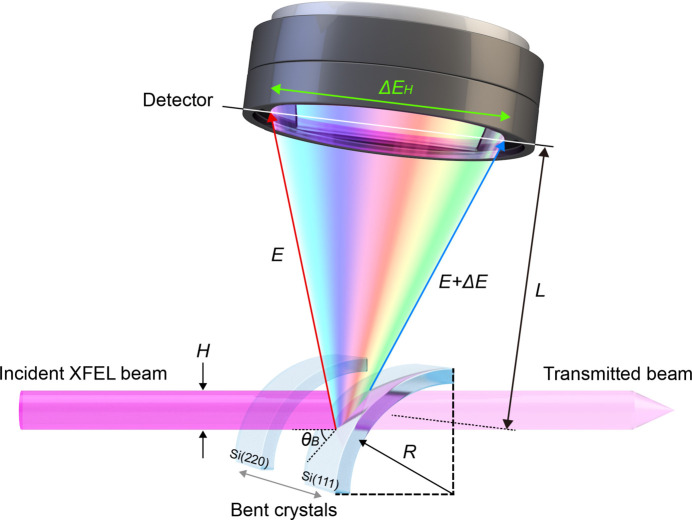
Schematic of the single-shot spectrometer at the hard X-ray beamline of the PAL-XFEL. The spectrometer adopts a 10 µm-thick Si crystal bent to a 100 mm radius of curvature. The distance between the bent crystal and detector is fixed at 1100.5 mm. This geometric configuration is consistently applied in both the calculation and experimental setup.

When a highly collimated X-ray beam with a finite bandwidth, such as an XFEL, is irradiated onto a thin, bent crystal, the incident angle with respect to the surface of the bent crystal varies depending on the irradiated position, and diffraction occurs at different exit angles. Thus, the diffracted beams from the thin, bent crystal form an angular spectrograph that can be recorded by a detector located at an appropriate distance. Two significant dispersion relationships for a bent crystal can be derived from its dispersion geometry, as shown in Fig. 1 ▸: (1) pixel energy resolution at the detector plane, expressed by
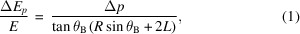
where *E* is the photon energy, 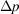 is the detector pixel size, θ_B_ is the Bragg angle, *R* is the radius of curvature of the thin, bent crystal, and *L* is the distance between the bent crystal and detector; (2) spectral range of the incident beam, which can be diffracted using a bent crystal,
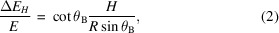
where *H* denotes the beam size. At a fixed radius of curvature of a bent crystal, the pixel energy resolution can be determined using a detector setup, whereas the spectral range can be specified by the beam size.

**Figure 2:**
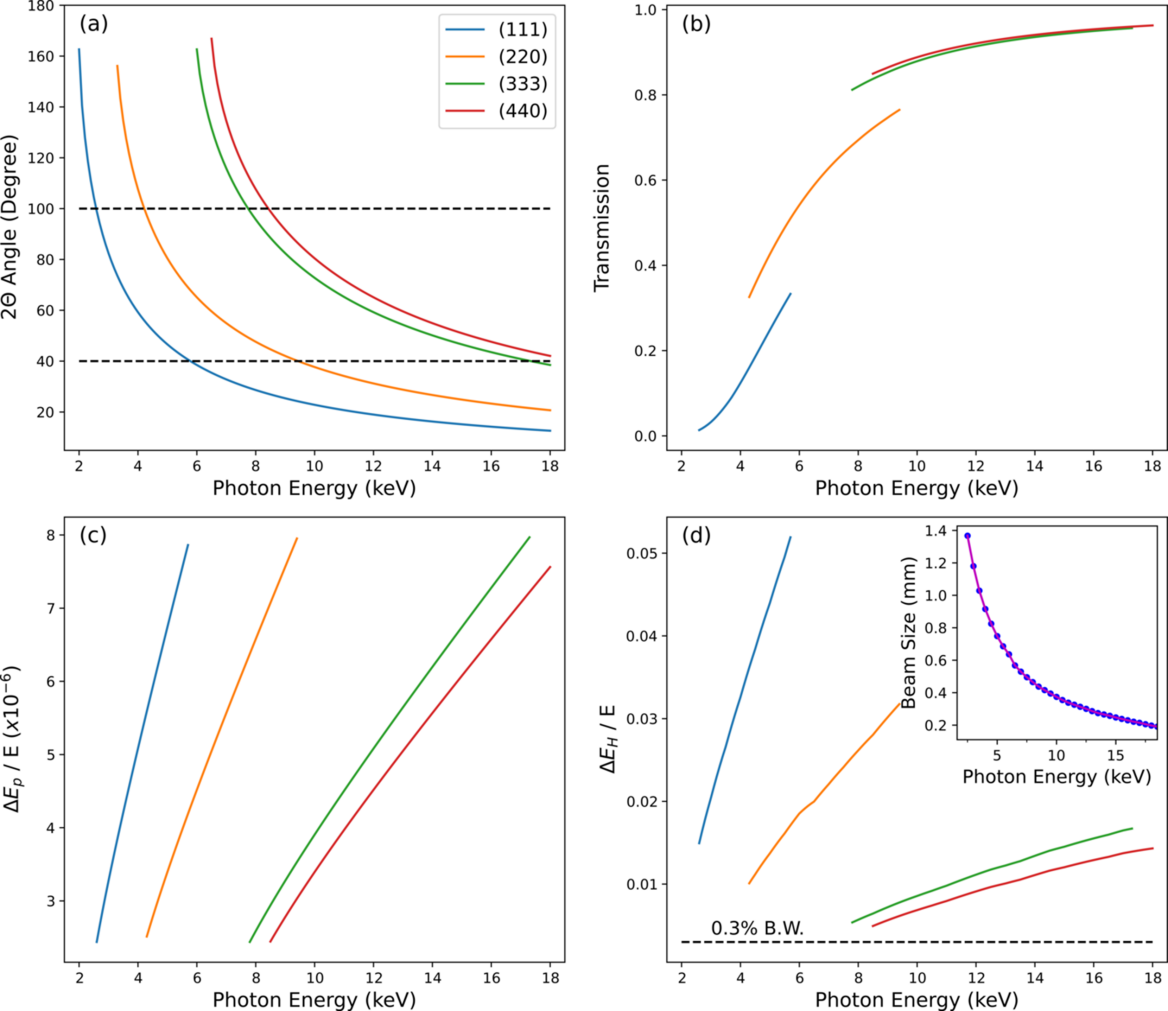
Design specification of the single-shot spectrometer. The available Bragg reflections are (111), (220), (333) and (440). The (*a*) 2θ (detector arm angle), (*b*) transmission, (*c*) 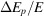 (pixel energy resolution) and (*d*) 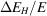 (measurable spectral range) parameters are calculated as functions of photon energy for each available Bragg reflection. In (*a*), the two dashed lines indicate the available angular range (2θ) defined by the Be window’s size. The technical specifications are calculated within the allowed range of 2θ. In (*d*), 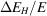 is determined by the beam size (FWHM) at the spectrometer’s location, which is calculated based on a previous study (Parc *et al.*, 2014 ▸).

The design specifications of the single-shot spectrometer have been determined based on these dispersion relationships (Fig. 2 ▸). In this calculation, the spectrometer is assumed to adopt a 10 µm-thick Si crystal bent to a 100 mm radius of curvature. The basic requirements for a single-shot spectrometer that can be installed in the hard X-ray beamline of the PAL-XFEL are as follows: (1) it should cover the photon energy range 4.5–17 keV; (2) its spectral range should be wider than the bandwidth of the SASE beam in the hard X-ray regime; (3) the pixel resolution should be smaller than 8 × 10^−6^ to achieve a high spectral resolution; (4) its instrumentation should be compact and simple for both easy operation and maintenance.

**Figure 3:**
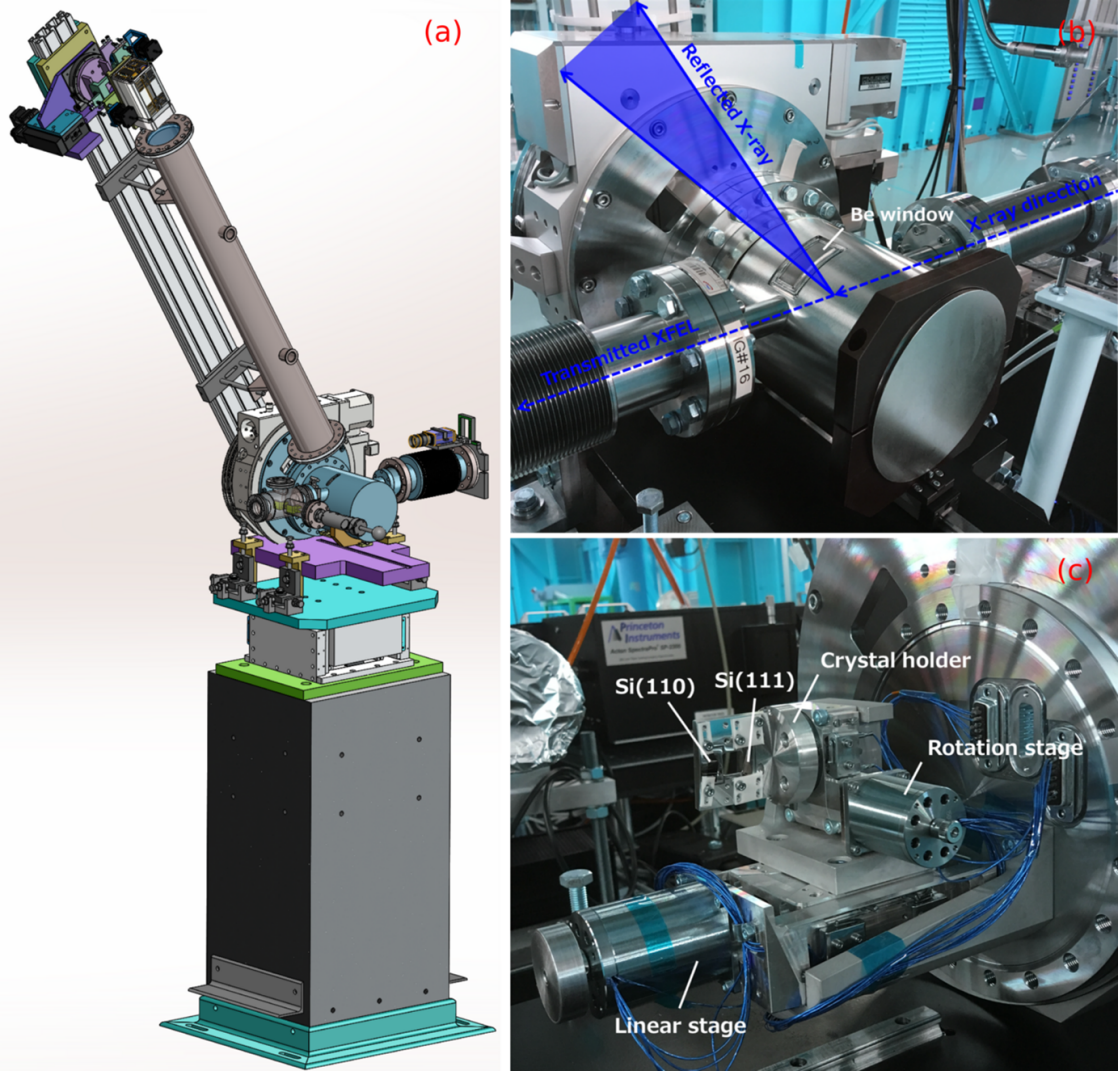
Single-shot spectrometer established at the hard X-ray beamline of PAL-XFEL. (*a*) Schematic of the single-shot spectrometer. (*b*) Outside and (*c*) inside view of the spectrometer’s chamber.

Considering these factors, a hard X-ray single-shot spectrometer has been developed (Fig. 3 ▸). The instrument consists of a crystal as a dispersive element, a vacuum chamber and a detector as a spectrum recorder. In the crystal part, to utilize the reflection of Si (111), (220), (333) and (440) according to photon energy, two crystals of Si (111) and Si (110) are mounted on the crystal holder and can be selected. If neither crystal is used, then a hole is provided through which a direct beam can pass. The crystal holder is mounted on a rotating stage that can change the Bragg angle θ and a linear stage for crystal selection and bypass. To achieve high transmission with minimal perturbation, a 10 µm-thick Si crystal, with crystal foil dimensions of 5 mm × 15 mm, has been utilized as a bent crystal. The crystals are mounted onto a crystal holder with a bending radius of 100 mm. In particular, in the photon energy range 4.5–17 keV, the spectrometer is designed to cover a spectral range that is wider than the full SASE bandwidth, guaranteeing sufficient resolution to resolve each spectral spike. In the photon energy range below 4.5 keV, the Si (111) reflection can be used for the single-shot spectrometer, which is not suitable for non-invasive measurement due to its low transmittance.

The vacuum chamber is mounted on a granite support to minimize the effects of vibration and is installed on a motorized stage that allows height adjustment of the entire chamber. A Be window is welded into the chamber to allow exit of the beam reflected by Bragg diffraction from the crystal. This Be window has been prepared using a very high purity Be foil (purity: 99.5%) with a thickness of 250 µm and width of 10 mm, in a direction perpendicular to the incident X-rays, to allow exit of the beam from the chamber. A Be window with a length of approximately 76.2 mm is used for the maximum possible duration in the direction in which the diffracted beam spreads. To use a wider 2θ range for the same-sized Be window, a small chamber with a diameter of approximately 8 inches has been designed. In addition, the θ–2θ rotation center has been located closer to the welded Be window than to the center of the chamber diameter to cover a large 2θ angle even with the length-limited Be window. It has a clear aperture of ∼30° based on the center of the chamber; however, it has been designed to feature a clear aperture range of ∼60°, from 40° to 100°, based on the 2θ of the crystal.

The detector, which is mounted on a 2θ arm driven by a goniometer, uses a scientific complementary metal–oxide–semiconductor (namely, sCMOS) camera with a pixel size of 6.5 µm and pixel number of 2560 × 2160. The position of the detector can be manually adjusted, on the 2θ arm up, to 1.2 m from the Si crystals. A detachable vacuum path has been inserted between the vacuum chamber and detector to enable the measurement of photon energy ranges exhibiting high absorption in air.

## 3. Results

**Figure 4:**
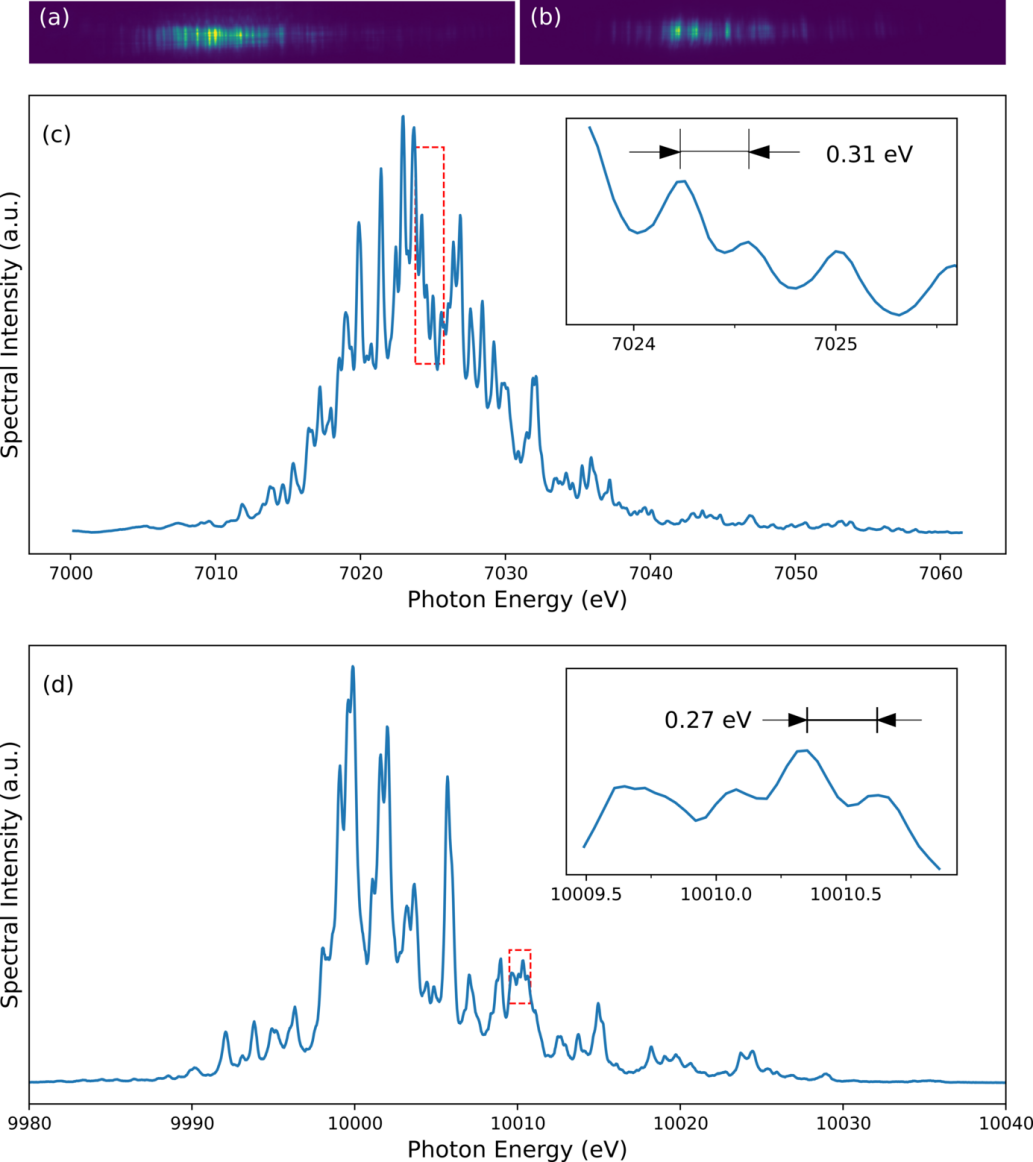
Single-shot spectra of a SASE beam. The spectra measurements are performed at *E* = 7.0 keV with the Si (220) reflection (*a*) and at *E* = 10 keV with the Si (333) reflection (*b*). Furthermore, to characterize the spectral properties of the SASE beam, the 2D spectral images [(*a*) and (*b*)] are integrated with the 1D spectra profiles [(*c*) and (*d*)]. The regions, indicated by the red dashed boxes, are enlarged and displayed in the corresponding inset figures to demonstrate the achieved spectral resolution of the single-shot spectrometer in the hard X-ray beamline of PAL-XFEL.

To evaluate the performance of the established spectrometer, both the measurable spectral range and spectral resolution have been investigated using single-shot spectra of the SASE beam, as shown in Fig. 4 ▸. To determine the achievable spectral range, spectral distributions are captured from the Si (220) and Si (333) reflections at *E* = 7.0 and 10.0 keV, respectively. As expected from the design specifications, the established spectrometer with thin Si crystals can provide a sufficiently wide spectral range to cover the full bandwidth of the SASE beam at various photon energies. Despite spectral fluctuations in the SASE beam, the entire spectral structure has been successfully monitored without any disturbance for both the Si (220) and Si (333) crystals.

To determine the energy resolution of the spectrometer, the minimum peak separation is measured, as shown in Figs. 4 ▸(*c*) and 4 ▸(*d*). In this case, the smallest peak separation is assumed to be the spectral resolution of the spectrometer (Makita *et al.*, 2015 ▸; Rich *et al.*, 2016 ▸). Thousands of SASE shots are collected and investigated to quantify the instrument’s energy resolution. The entire spectrum is fitted to identify all the local maxima from which resolvable peak separations can be derived. The achieved spectral resolutions for the Si (220) and Si (333) thin crystals are smaller than 0.31 and 0.27 eV at *E* = 7.0 and 10 keV, respectively, indicating that the resolving powers of these crystals are higher than 2.3 × 10^4^ and 3.7 × 10^4^, respectively.

**Figure 5:**
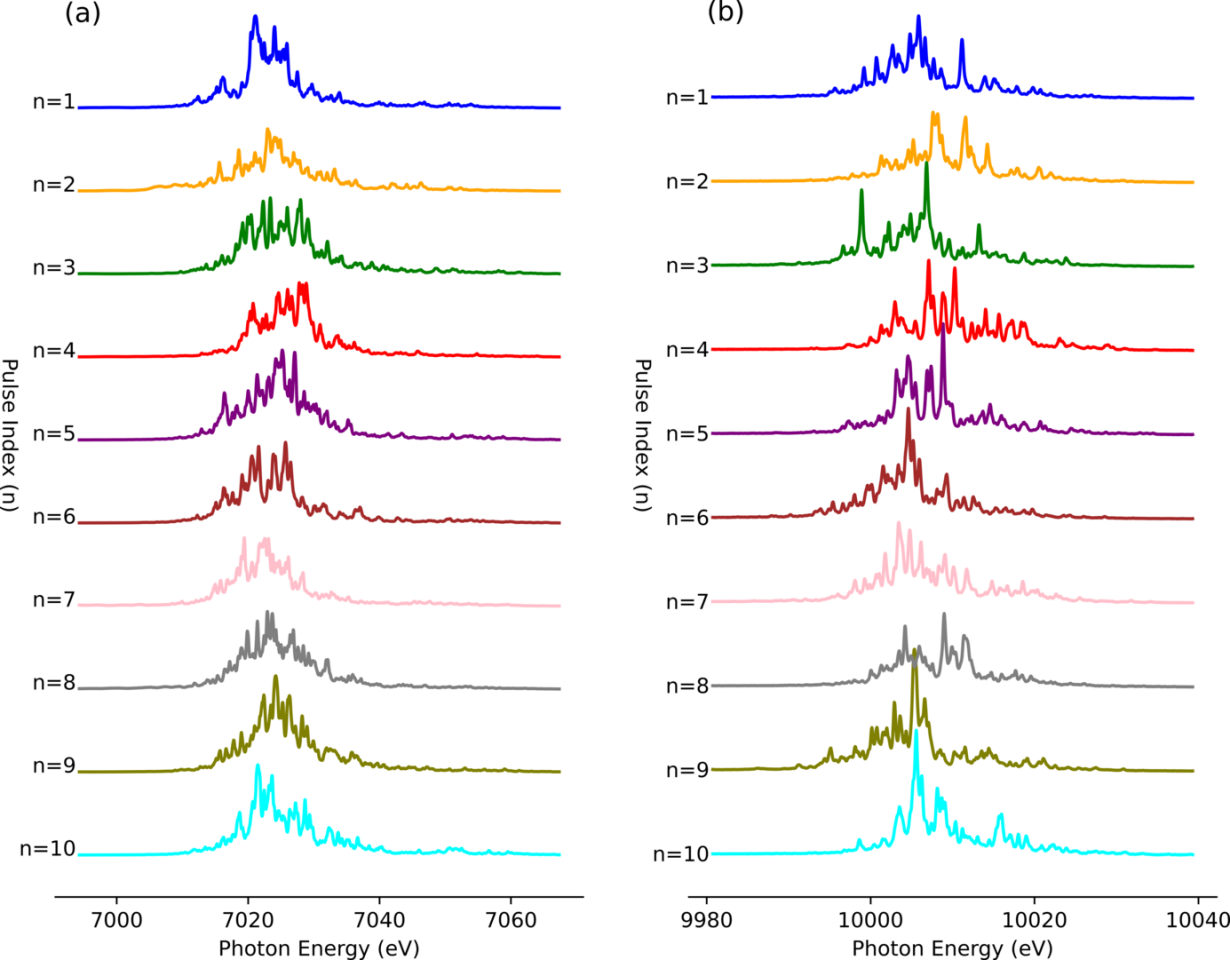
SASE spectra of ten consecutive XFEL pulses captured from (*a*) Si (220) at *E* = 7.0 keV and (*b*) Si (333) at *E* = 10.0 keV, showing fluctuations both in peak intensity and spectral distribution.

To reveal the stochastic nature of the SASE beam, one-dimensional (1D) spectral images of the thin Si crystals are analyzed (Fig. 5 ▸). The SASE spectra of typically ten consecutive XFEL pulses are captured from Si (220) and Si (333) at *E* = 7.0 and 10.0 keV, respectively. As expected from XFEL theory, each pulse spectrum shows both intensity and distribution fluctuations (Saldin *et al.*, 1998 ▸). These shot-to-shot fluctuations correspond to the fingerprints of the entire SASE process, justifying the establishment of the single-shot spectrometer. To date, the shot-to-shot analysis method, which provides essential information for single-shot-based experiments, has been extensively investigated. However, a statistical approach is necessary to optimize and characterize the beam conditions of XFEL sources.

**Figure 6:**
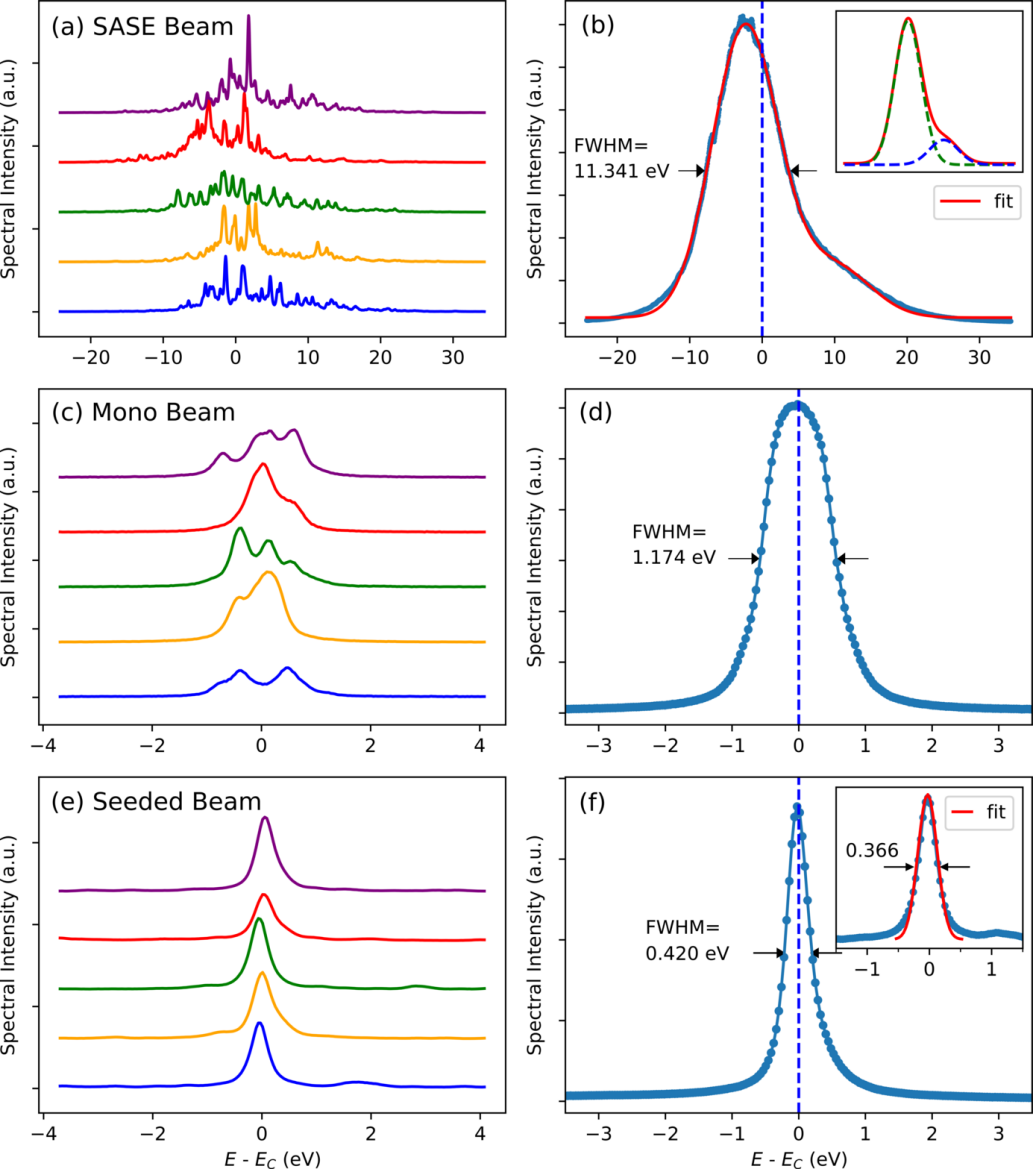
Typical spectral distribution in a (*a*, *b*) SASE beam at *E*_c_ = 10004.97 eV, (*c*, *d*) monochromatic beam of SASE pulses at *E*_c_ = 10000.06 eV and (*e*, *f*) seeded beam at *E*_c_ = 9996.16 eV. The Si (333) spectrometer is utilized to measure the spectral distribution, and the monochromatic beam is prepared by passing the SASE pulses through a Si (111) DCM. The first column of the figure [(*a*), (*c*) and (*e*)] reveals shot-to-shot spectral fluctuations in the XFEL pulses; the second column [(*b*), (*d*) and (*f*)] shows the average profile of 1000 consecutive shots. However, a single-shot spectrum [inset of (*f*)] is also investigated, especially for the seeded beam, to obtain a more precise bandwidth of the seeded beam.

Depending on the experimental requirements, the hard XFEL beamline at the PAL-XFEL can provide a variety of XFEL sources, including SASE beams, monochromatic beams of the SASE mode with a Si (111) double-crystal monochromator (DCM), and seeded beams (Kim *et al.*, 2018 ▸; Nam *et al.*, 2021 ▸). In this study, an established spectrometer has been used to characterize the spectral properties of these sources. Fig. 6 ▸ shows the spectral distributions of the SASE, monochromatic and seeded beams obtained using the *in situ* single-shot and statistical multiple-shot methods. All the spectra have been measured using a single-shot spectrometer equipped with a Si (333) bent crystal. Fig. 6 ▸(*a*) shows shot-to-shot SASE spectra captured in the real-time mode, revealing the stochastic spectral properties. To statistically characterize the spectral distribution of the SASE beam, the average profile of 1000 consecutive shot spectra is analyzed, as shown in Fig. 6 ▸(*b*). As shown in the inset of Fig. 6 ▸(*b*), two Gaussian curves can fit the measured SASE profile such that the centroid (*E*_CEN_) and full width at half-maximum (FWHM) of the large Gaussian curve are *E*_CEN_ = 10002.64 eV and FWHM = 10.798 eV, respectively, whereas those of the small curve are *E*_CEN_ = 10015.55 eV and FWHM = 14.130 eV, respectively. The fitting results indicate that two Gaussian sources contribute to the overall spectral distribution of the SASE beam during 1000 consecutive shots, and the corresponding bandwidth of the SASE beam is 11.341 eV (0.11%) at *E* = 10 keV. The small Gaussian source increases the overall bandwidth by 0.55 eV, and another Gaussian distribution has a relatively small effect on the SASE bandwidth owing to its low amplitude. However, depending on the beam tuning conditions, this type of source can be enhanced, and the SASE bandwidth can be increased. Therefore, careful monitoring of the single-shot spectrometer is essential to optimize the bandwidth of the SASE beam.

The SASE beam traversing the Si (111) DCM yields a narrow spectral distribution that is adequate for high-energy-resolution spectroscopy. Fig. 6 ▸(*c*) shows several representative monochromatic spectra acquired in this study. The Darwin width of the Si (111) DCM at *E* = 10 keV is 37.6 µrad (*X-ray Server*, https://x-server.gmca.aps.anl.gov/) and the corresponding spectral width is 1.865 eV. Because the Si (111) DCM operates as a bandpass filter, only a few spikes from the SASE beam traversing the DCM survive. The observed number of resolvable spikes in the monochromatic beam spectra is approximately four because of the resolution limit of the Si (333) spectrometer [Fig. 4 ▸(*d*)]. The average profile of the 1000 shots monochromatic spectra is shown in Fig. 6 ▸(*d*). The measured bandwidth of the monochromatic beam at *E* = 10 keV is 1.174 eV (0.012%), which is smaller than the Darwin width of the Si (111) DCM (1.865 eV). This result implies that the spectral spikes selected from the SASE beam are distributed over an extremely narrow range instead of spreading over the Darwin width.

Single-shot spectrometry is an essential diagnostic tool that provides high-quality seeded beams for XFEL applications. The seeded beam can be optimized by observing and tuning the beam parameters to enhance the seeded beam spectra in real-time. The PAL-XFEL can provide a high-quality self-seeding beam by leveraging the forward Bragg diffraction through a thin diamond crystal (Nam *et al.*, 2021 ▸). In this study, a 100 µm-thick diamond crystal with a [100] cut and Bragg reflection plane of (115) was used to generate a seeded beam at a photon energy of 10.0 keV. Fig. 6 ▸(*e*) shows several images of the seeded beam spectra obtained in our study. Owing to the resolution limit of the spectrometer, fine spectral structures cannot be resolved. The average profile of 1000 consecutive shots is shown in Fig. 6 ▸(*f*). Unlike the monochromatic beam spectra, the tail of the seeded beam spectrum deviates from the Gaussian distribution. Consequently, Gaussian fitting can only be applied to the central peak to characterize the seeded beam spectra. The apparent bandwidth of the seeded beam at *E* = 10 keV is 0.42 eV (relatively 0.0042%). However, because the bandwidth of the seeded beam is comparable with that of the resolution function of the instrument (spectrometer), deconvolution of the resolution function may be necessary. As shown in Fig. 4 ▸(*d*), the resolution of the spectrometer comprising the Si (333) bent crystal is smaller than 0.27 eV. Thus, deconvolution of the resolution function can reduce the actual bandwidth to ∼0.32 eV. Although any existing spike within the central peak of the seeded beam cannot be properly resolved using the established spectrometer, simple monitoring of the seeded beam spectra can provide sufficient information to optimize the beam parameters. Owing to the extremely narrow bandwidth of a seeded beam, the spectral fluctuations between XFEL pulses can considerably broaden the apparent bandwidth. Consequently, a single-shot approach is required to obtain the precise bandwidth of a seeded beam. The inset of Fig. 6 ▸(*f*) shows an example of a single-shot approach. The apparent bandwidth measured at *E* = 10 keV is 0.366 eV, which can be reduced to ∼0.25 eV after deconvolution of the spectrometer’s resolution. Notably, more accurate measurements have been performed using Si (333) flat crystals, and a bandwidth of 0.19 eV at *E* = 9.7 keV has been reportedly obtained (Nam *et al.*, 2021 ▸).

**Figure 7:**
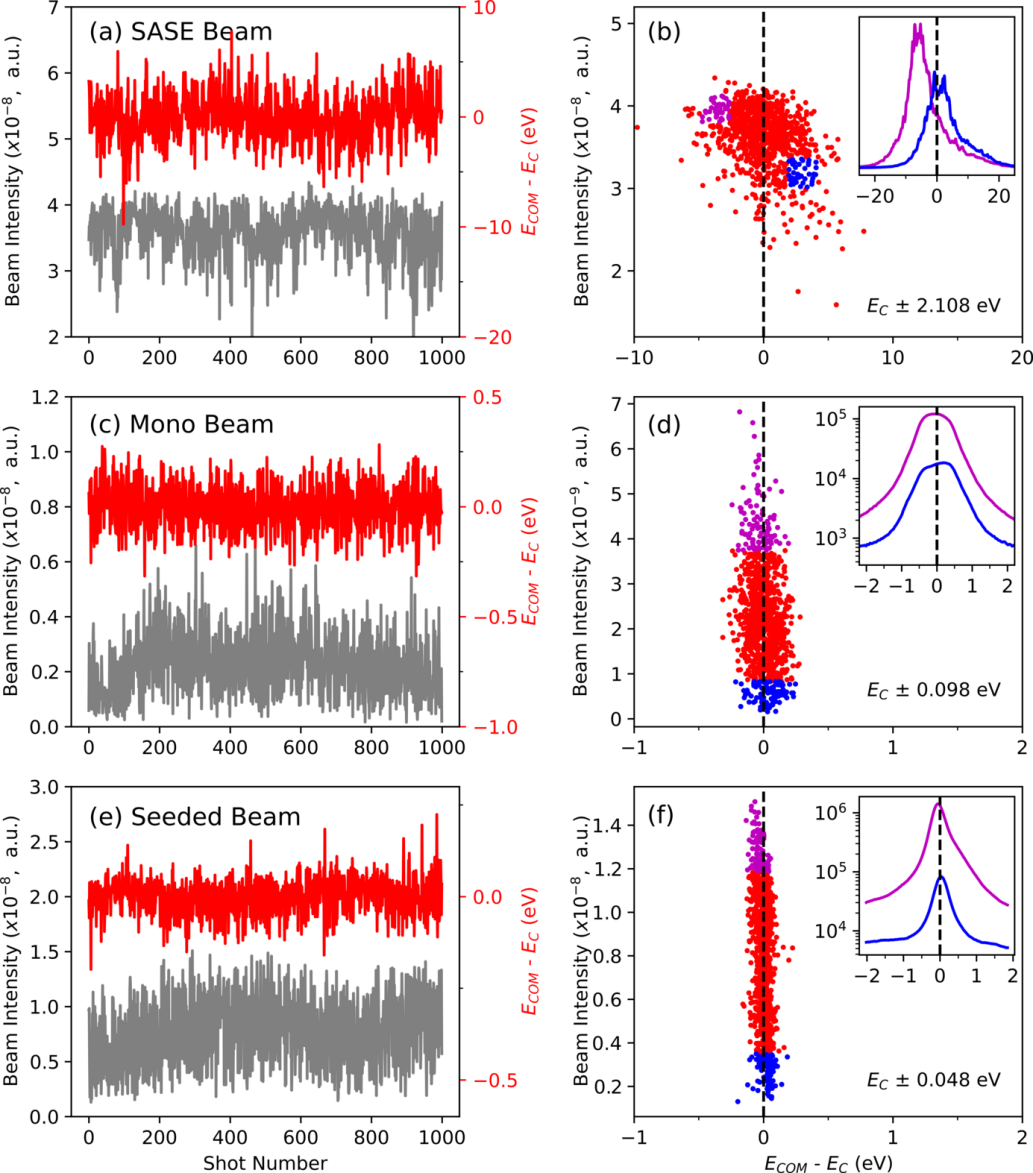
Typical spectral fluctuations in a (*a*, *b*) SASE beam at *E*_c_ = 10004.97 eV, (*b*, *c*) monochromatic beam of SASE pulses at *E*_c_ = 10000.06 eV, and (*e*, *f*) seeded beam at *E*_c_ = 9996.16 eV. The first column of the figure [(*a*), (*c*) and (*e*)] shows fluctuations in peak position and intensity; the second column [(*b*), (*d*) and (*f*)] represents the correlation between the intensity and position of the XFEL pulses. In particular, two representative regions (blue and magenta points) are selected, and the average profiles of the two regions are shown in each inset of the second column [(*b*), (*d*) and (*f*)] with the same color of the corresponding two regions.

In addition to the fluctuations in spectral distributions, those in both peak position and intensity characterize the statistical properties of shot-to-shot spectra. The beam intensity is monitored by a beamline diagnostic device, called a quadrant beam position monitor. The fluctuations in the peak position can be easily monitored by examining the center of mass (COM) of a single-shot spectrum. The data presented in Fig. 6 ▸ have been used to calculate the COM of the single-shot spectrum and compare it with the beam intensity. The first column of Fig. 7 ▸ shows both the COMs and beam intensities independently in the corresponding beam modes. The measured relative root-mean-square (RMS) intensity fluctuations, defined by 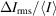, for the SASE, monochromatic and seeded beams are 10.1%, 50.7% and 39.7%, respectively; the corresponding calculated COM fluctuations, or standard deviations of the COMs, are 2.108, 0.098 and 0.048 eV, respectively. As expected, the intensity fluctuation is inversely proportional to the average intensity of the beam sources. Moreover, the measured bandwidths depicted in Fig. 6 ▸ are correlated with the measured COM fluctuations presented in Fig. 7 ▸. Evidently, the COM fluctuations tend to increase when the bandwidth of the XFEL source is increased.

To further examine the spectral fluctuations, the correlations between the beam intensity and COM have also been investigated; Fig. 7 ▸(*b*) illustrates this correlation for the SASE beam. Two representative regions (blue and magenta) are selected to compare the spectral properties, and the average spectral profiles of the two regions are shown in the inset of Fig. 7 ▸(*b*) using the same colors of the two regions. The scatter plot shows an oblique elliptical shape, implying that the COM is inversely correlated with the beam intensity; in other words, the higher the beam intensity, the lower the COM energy (*E*_c_), and vice versa. The most intense SASE sources are located below *E*_c_, consistent with the fitting results shown in Fig. 6 ▸(*b*). Thus, the intensity fluctuations may arise from two different mechanisms, viz. the stochastic fluctuation of the SASE beam and change in the preferred SASE mode. The intensity fluctuation along the vertical direction in Fig. 7 ▸(*b*) indicates the stochastic fluctuation, whereas that along the oblique direction (from the blue region to the magenta region) shows a change in the preferred SASE source as well as the stochastic nature of the SASE beam. To gain insights into the spectral features, information about the spatial fluctuations in the beam may be necessary. A bent crystal spectrometer maps portions of the XFEL beam profile to different energies, implying that spatial fluctuations in the XFEL beam can result in spectral fluctuations in the measured spectra. For instance, the two Gaussian peaks in Fig. 6 ▸(*b*) might have originated from the spatial fluctuations in the SASE beam. Similarly, the intensity fluctuations along the horizontal direction in Fig. 7 ▸(*b*) could be related to these spatial variations. Thus, simultaneous monitoring of spatial fluctuations is recommended to ensure accurate spectral interpretation.

In contrast, the oblique elliptical type of correlation cannot be applied to monochromatic and seeded beams. Because a crystal acts as a bandpass filter, its Darwin width defines the available range of the COM. The crystal accepts only a few spikes from the single-shot spectra of the SASE beam. Therefore, the beam intensity does not correlate with the COM, as shown in Figs. 7 ▸(*d*) and 7 ▸(*f*). Additionally, the fluctuations in the monochromatic and seed beam intensities are stronger than those in the SASE beam. To explain these results in detail, we assume that the monochromatic beam is optimized at the peak position of the magenta curve shown in the inset of Fig. 7 ▸(*b*). For example, if the status of the SASE beam changes from the magenta curve to the blue curve, then its intensity fluctuates because the integrated intensity over its spectral range changes. A mere change in the peak position does not guarantee the occurrence of intensity fluctuations; rather, it reflects a change in the integrated intensity. By contrast, the DCM crystal, which is optimized at the peak position of the magenta curve shown in Fig. 7 ▸(*b*), can only accept the tail of the blue curve depicted in Fig. 7 ▸(*b*). Thus, compared with SASE beams, monochromatic beams are more susceptible to spectral fluctuations. According to the measured RMS fluctuations, the fluctuations in the monochromatic beam intensity are five times higher than those in the SASE beam intensity. The same mechanism is valid for seeded beams; however, the fluctuations in seeded beam intensity are four times higher than those in SASE beam intensity.

## 4. Conclusion

We have developed a transmissive single-shot spectrometer to monitor the shot-to-shot spectral structures in the hard X-ray beamline of the PAL-XFEL. The spectrometer comprises 10 µm-thick Si crystals that are bent to a 100 mm radius of curvature. Depending on the photon energy, either the Si (111) crystal or Si (110) crystal is selected for the experiment. This instrument can cover a photon energy range of 4.5–17 keV, providing a spectral range wider than the full FEL bandwidth of the photon energy. We have achieved a resolving power of *E*/δ*E* > 3.7 × 10^4^ at *E* = 10.0 keV with the Si (333) spectrometer. The established spectrometer, which can be applied to any XFEL experiment that requires information on the spectral structure of each pulse for analyzing the experimental data, is an essential diagnostic tool for operating the hard XFEL beamline of the PAL-XFEL. Moreover, it can be utilized to characterize as well as optimize the XFEL operating conditions. As an example, we have used the developed spectrometer to evaluate the spectral properties of various beam modes, including SASE, monochromatic and seeded beams. First, single-shot spectra of the XFEL pulses are captured to assess the stochastic nature of the XFEL sources. Subsequently, the average spectral distributions of the XFEL sources are examined to measure their bandwidths and spectral ranges. Finally, the correlation between the intensity and position of the XFEL pulses is evaluated to explain the dependence of the intensity fluctuations on the XFEL sources. In summary, the spectrometer can efficiently monitor single-shot spectra recorded at the hard XFEL beamline of PAL-XFEL.

## Data Availability

Data underlying the results presented in this paper are not publicly available at this time but may be obtained from the authors upon reasonable request.
